# Neutral endopeptidase inhibitor suppresses the early phase of atrial electrical remodeling in a canine rapid atrial pacing model

**Published:** 2008-04-01

**Authors:** Ryuta Imaki, Shinichi Niwano, Hiroe Niwano, Daisuke Satoh, Toru Yoshida, Yoshihiko Masaki, Tohru Izumi

**Affiliations:** Department of Internal Medicine/Cardiology, Kitasato University School of Medicine, Sagamihara, Japan

**Keywords:** electrical remodeling, atrial natriuretic peptide, neutral endopeptidase inhibitor, cyclic GMP, calcium

## Abstract

**Introduction:**

We examined the acute effects of neutral endopeptidase inhibitor on the hemodynamics and electrical properties of dogs subjected to rapid atrial pacing.

**Methods:**

Ten beagle dogs were used and divided into two groups with and without candoxatril, a neutral endopeptidase inhibitor preadministration.  Before and after the 6 hours rapid atrial pacing from the right atrial appendage, the hemodynamics, atrial effective refractory period, and monophasic action potential duration of the right atrial appendage were measured and blood samples were collected.  Atrial tissue was also excised after the experiment.

**Results:**

Candoxatril significantly increased plasma ANP levels (Control: 88.4 ± 50.25 vs. Candoxatril: 197.1 ± 32.09 pg/ml, p = 0.004) and prevented reductions in atrial effective refractory period and monophasic action potential duration.  We further demonstrated that the treated animals exhibited significantly higher levels of atrial tissue cyclic GMP (Control: 28.1 ± 1.60 fmol/mg vs. Candoxatril: 44.5 ± 12.28 fmol/mg, p = 0.034) as well as that of plasma cyclic GMP (Control: 32 ± 5.5 vs. Candoxatril: 42 ± 7.1 pg/ml, p = 0.028).

**Conclusion:**

Candoxatril suppressed the shortening of atrial effective refractory period and monophasic action potential duration in the rapid atrial pacing model.  As plasma ANP and the atrial tissue levels of cyclic GMP were higher in the Candoxatril group than the control, this effect was considered to appear through the reduction of calcium overload caused by ANP and cyclic GMP.

## Introduction

Atrial fibrillation (AF) is a common arrhythmia, especially in elderly individuals with cardiac disorders.  Atrial electrical remodeling, characterized by shortening of the effective refractory period (ERP) and decreased conduction velocity, is considered to play an important role in the development of the AF substrate [1].  According to several reports, intracellular calcium overload seems to be the initial key to promote atrial electrical remodeling, especially in its early phase, and several interventions reducing calcium overload, such as the L-type calcium blocker and the sodium channel blocker, have been shown to prevent the initial phase of atrial electrical remodeling [2-5].

We have previously reported that human atrial natriuretic peptide (hANP) suppresses the early phase of atrial electrical remodeling in a canine rapid atrial pacing model [6]. Atrial natriuretic peptide (ANP) is produced principally in the atrium and is secreted in response to mechanical strain on the heart muscle.  It is reported that actions of ANP are mediated by increase in intracellular cyclic GMP after ANP binding to its guanylate cyclase-linked cell surface receptors [7,8]. Elevation in cyclic GMP decreases myocyte contraction, and this effect is mainly mediated through the cyclic GMP-dependent protein kinase and can reduce intracellular calcium [9,10].  Therefore ANP would be expected to suppress the calcium overload through the action of cyclic GMP signaling.  Although this preventive effect of ANP against the initiation of atrial electrical remodeling is considered reasonable to maintain homeostasis, ANP has a significant limitation in clinical use because it cannot be orally administered. In this study, the effect of candoxatril (Cx), a neutral endopeptidase inhibitor (NEPI), on atrial electrical remodeling was studied.  Because neutral endopeptidase is a membrane-bound metalloenzyme that cleaves endogenous peptide such as ANP [11,12], we hypothesized that oral administration of Cx would potentiate the role of endogenous ANP and suppress the electrical remodeling.

## Methods

### Subjects and preparation

Ten beagle dogs weighing 12.5 to 22.1 kg were used for this study, and the preparation followed our previous reports [13-15].  Initial anesthesia was made with an intravenous injection of pentobarbital (25 mg/kg), and it was kept with an additional administration of pentobarbital (3 mg/kg/hr) after intubation and mechanical ventilation (Harvard Inc., Boston, MA, USA).  Tidal volume and oxygen concentration were adjusted to maintain arterial pH between 7.35 and 7.45, and the heart was exposed via a right thoracotomy.  After an 8F sheath was placed in the right femoral vein, a radiofrequency ablation catheter was inserted and the His bundle was ablated to avoid rapid ventricular response during rapid atrial pacing (Marinr MC, 4mm tip, 7F and Atakr, Medtronic Japan Inc, Tokyo, Japan).  After His bundle ablation, the ventricular rate was maintained by epicardial ventricular pacing at 100 beats per minute (THERA SR-i 8960i Medtronic Japan Inc., Tokyo, Japan).  Systemic blood pressure was monitored continuously from the femoral artery, and central venous pressure and pulmonary capillary wedge pressure were measured by a Swan-Ganz catheter introduced through the femoral vein.  A pair of stainless steel wire electrodes was fixed at the epicardial surface of the right atrial appendage (RAA), and this was used for continuous rapid atrial pacing.  Two pairs of platinum plunge-wire electrodes were fixed at the right atrial surface of the pectinate muscle and were used to evaluate the atrial effective refractory period (AERP) and the monophasic action potential duration (MAPD).  In this study, we used hook-shaped customized needle-electrodes for pacing and recording [16].  They were punctured on the surface of the RAA and sutured to keep secured position.  All studies were performed in accordance with the guidelines specified by the Animal Experimentation and Ethics Committee of Kitasato University School of Medicine.

### Drug administration

Candoxatril (Cx; Pfizer Co., Ltd., London, GB) was used as a neutral endopeptidase inhibitor in this study. In 5 of 10 dogs, 24 mg/kg of Cx at 12 hours and 20 mg/kg of Cx at 2 hours before the experiment were orally preadministered (Cx group).  Because Cx is not commercially available and the amount of it was totally limited, only single dose of Cx which concentration in plasma could not be measured was examined. But a proper dose of Cx was determined from the previous report [17] to achieve plasma levels of inhibitor exceeding IC_95_ that is the concentration at which 95 % of biological activity is inhibited throughout the experiments. The remaining 5 dogs without Cx administration were evaluated as the control.

### Pacing protocol and electrophysiological measurement

After the initial surgical procedure, the dogs were allowed to recover for 60 minutes to achieve a stable state, and AERP and MAPD were evaluated for three different basic cycle lengths (BCL), i.e., 300, 200 and 150 ms.  Continuous rapid atrial pacing from RAA at 400 beats per minute was then started and continued for 6 hours (SEN-7203, Nihon-Kohden Co., Tokyo, Japan), and AERP and MAPD were re-evaluated after this 6-hour pacing. In the electrophysiological evaluation, stimulus was delivered at an output twice the diastolic threshold with a 2 ms rectangular pulse, the coupling interval of the extrastimulus was shortened by a 2 ms step, and the longest coupling interval which failed to capture the atrium was defined as AERP.  For the evaluation of MAPD, the interval between peak deflection and the 20% or 90% recovery point of the MAP trace was measured, i.e., MAP20 and MAP90, respectively.  In this study, we did not evaluate other parameters of the electrical remodeling such as the conduction time and the inducibility of AF.  Because early phase of the electrical remodeling is assumed to recover in short term [18], complicated protocol could cause the distrust of accuracy.

The surface ECG and atrial electrogram were amplified using a polygraph system (Polygraph 365, NEC Inc., Tokyo, Japan).  These analog signals were converted to digital signals with a sampling frequency of 1000 Hz (Power Lab 8 sp, Bioresearch Co., Ltd., Tokyo, Japan) and stored on a computer.  The band-pass filter was set at 50-300 Hz for the standard cardiac electrogram and open-300 Hz for MAP recording.

## Biochemical analysis of neuro-endocrinal factors and cyclic GMP

A blood sample was taken from the femoral artery before and after the rapid atrial pacing protocol to assay the plasma levels of neuro-endocrinal factors and cyclic GMP.  ANP, renin activity, angiotensin II (Ang II), aldosterone and cyclic GMP were measured by radio immunoassays (Sumikin Bio-Science Inc., Japan).  Right atrial tissue was excised at the end of the whole protocol, frozen in liquid nitrogen and stored at -80 °C to measure myocardial cyclic GMP.  Using a purified atrial tissue extract, an enzyme-immunoassay for cyclic GMP was performed according to the manufacturer's protocol (Amersham Biosciences, Corp. USA.).

### Statistical analysis

Values are presented as the mean ± SD.  A paired t-test was used for the continuous values and ANOVA was used for comparison between groups of discrete variables.  Statistical significance was defined as P<0.05.

## Results

### Hemodynamics

Table 1 shows the hemodynamic parameters during the study protocol. Significant differences did not appear during the time course and there were no significant differences between the two groups. This indicates that NEPI did not affect the hemodynamics in this study protocol.

### Atrial effective refractory period (AERP)

**Figure 1:**
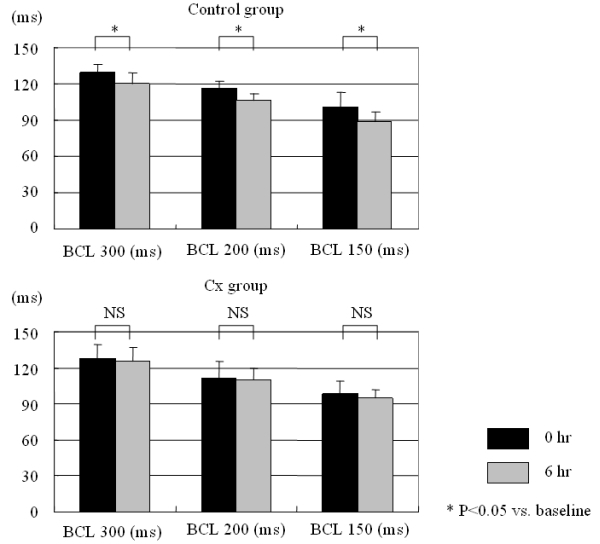
Changes in AERPs from a rapid pacing protocol.  In the control group, AERPs were shortened significantly by rapid atrial pacing at all basic cycle lengths (BCLs).  In contrast, in the candoxatril group, this AERP shortening was suppressed and no significant difference was observed.  See text for discussion. * p<0.05, NS: not significant.

AERP data are shown in Figure 1. In the control group, AERPs were shortened significantly at all basic cycle lengths (BCL300: from 130 ± 7 to 120 ± 10 ms, p = 0.043, BCL200: from 116 ± 6 to 106 ± 7 ms, p = 0.003, BCL150: from 101 ± 12 to 88 ± 8 ms, p = 0.022). In contrast, in the Cx group, AERP shortening was suppressed and no significant difference was observed before and after the rapid pacing.

### Monophasic action potential duration (MAPD)

**Figure 2:**
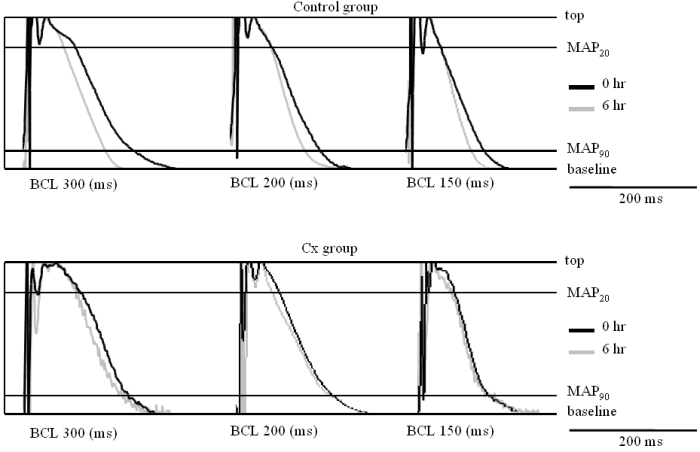
Representative traces of the monophasic action potential (MAP) of atrial electrograms.  The upper panels show the traces in the control group and the lower panels show those in the candoxatril group.  In each panel, the solid trace shows the recording at baseline (0 hr) and the gray line indicates the recording after 6 hr rapid pacing.  The horizontal bars indicate the measurement points of MAP20 and MAP90, respectively.  The shortening of the MAP duration was relatively obvious in the control group, whereas the change seemed to be much smaller in the candoxatril group.  See text for discussion. BCL: basic cycle length.

**Figure 3:**
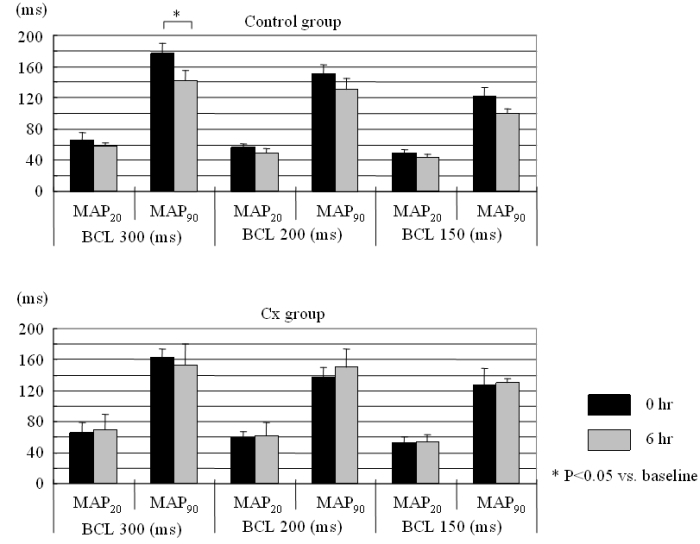
Changes in MAP20 and MAP90.  The upper panel shows the MAPD data in the control group and the lower panel shows those in the candoxatril (Cx) group.  Although the change demonstrated significance only in MAP90 at 300 ms basic cycle length (BCL), MAPD tended to be shortened in the control group.  In contrast, in the Cx group, no MAPD change tendency was observed.  See text for discussion.* P<0.05

Figure 2 shows representative MAP traces at three basic cycle lengths recorded at the RAA site.  In the control group, MAP traces showed relatively obvious shortening in their durations at all basic cycle lengths after the rapid pacing protocol, but there was no change in the Cx group.  Figure 3 shows MAPD data, i.e., MAP20 and MAP90, calculated from the MAP traces.  Although the change showed significance only in MAP90 at 300 ms BCL (from 177 ± 13 to 143 ± 12 ms, p = 0.021), MAPD tended to be shortened in the control group. In contrast, in the Cx group, no MAPD change tendencies were observed.

### Levels of neuro-endocrinal factors and cyclic GMP

**Figure 4:**
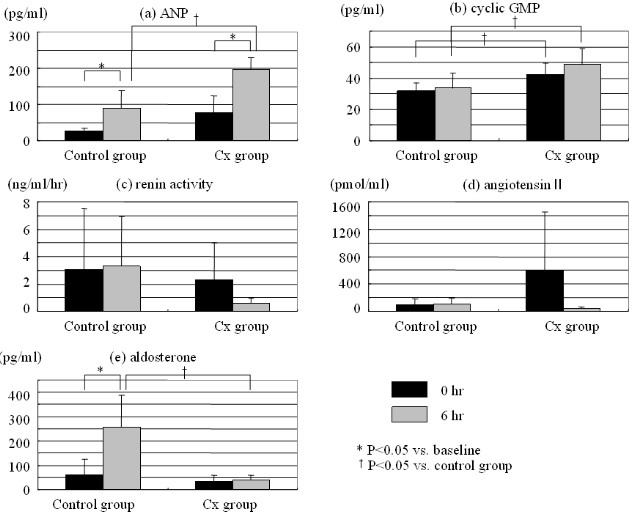
Changes in plasma concentrations of (a) ANP, (b) cyclic GMP, (c) renin activity, (d) angiotensin II and (e) aldosterone.  The ANP level was significantly increased by rapid pacing in both groups, and that of after the rapid pacing was higher in the Cx group than the control.  The level of cyclic GMP was not changed significantly by rapid pacing, but the level was higher in the Cx group than the control.  There was no difference between the two groups in renin activity and angiotensin II levels.  The level of aldosterone was increased in the control group but not in the Cx group.  See text for discussion.* p<0.05

Changes in plasma concentrations of ANP, cyclic GMP, renin activity, Ang II and aldosterone are shown in Figure 4. The plasma level of ANP was significantly increased by rapid pacing in both groups (control: from 25.6 ± 9.05 to 88.4 ± 50.25 pg/ml, p = 0.033, Cx: from 75.8 ± 48.63 to 197.1 ± 32.09 pg/ml, p = 0.014), and that of after rapid pacing was higher in the Cx group than the control (88.4 ± 50.25 vs. 197.1 ± 32.09 pg/ml p = 0.004). There was no difference between the two groups in renin activity and angiotensin II levels. The plasma level of aldosterone was increased in the control group (from 61 ± 63.2 to 256 ± 133.1 pg/ml, p = 0.028), but not in the Cx group after rapid pacing (from 32 ± 26.4 to 40 ± 20.6 pg/ml, p = 0.753).

**Figure 5:**
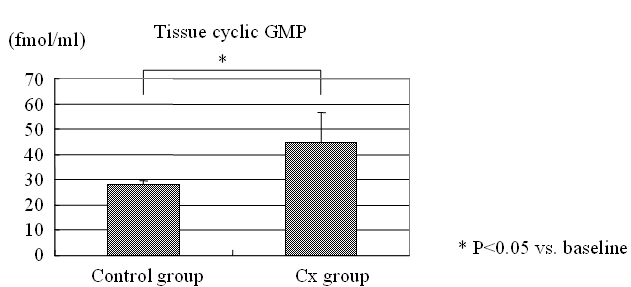
The level of tissue cyclic GMP after rapid pacing in the right atrial myocardium was significantly higher in the Cx group than the control.  See text for discussion.* p<0.05

On the other hand, the plasma level of cyclic GMP was not changed significantly by rapid pacing, but the level was higher in the Cx group than the control (baseline: 32 ± 5.5 vs. 42 ± 7.1 pg/ml, p = 0.028, after rapid pacing: 34 ± 9.9 vs. 48 ± 10.2 pg/ml, p = 0.048).  Additionally, the level of cyclic GMP in the extract of the right atrial myocardium was significantly higher in the Cx group than the control after rapid pacing (28.1 ± 1.60 vs. 44.5 ± 12.28 fmol/mg, p = 0.034) (Figure 5).

## Discussion

In this study evaluating the effect of Cx on atrial electrical remodeling in a canine rapid atrial pacing model, we noted several interesting findings: First, AERP shortening caused by rapid pacing was suppressed by Cx administration; second, there was no significant change in the hemodynamics; third, the plasma and tissue levels of cyclic GMP were higher in the Cx group than the control.  Finally, there was no significant change in the markers of the renin-angiotensin system in the circulating blood although an increase in aldosterone was observed after rapid pacing only in the control group.

### Effect of Cx on hemodynamics

Although Cx administration enhanced the plasma level of ANP which was known to improve the hemodynamics in the condition of heart failure, there was no significant difference in this study.  It is unclear why increased ANP did not cause any changes in the hemodynamics, but there were two conceivable reasons.  One is that because the effect of ANP has been usually documented in diseased hearts, its effect may be small in the normal hemodynamic condition as it was in this study.  In our previous study in which we demonstrated that intravenous administration of ANP suppressed the atrial electrical remodeling, there was also no change in the hemodynamics [6].  Another reason is that as neutral endopeptidase catalyzes the degradation of a number of endogenous vasodilator peptides, e.g., ANP, as well as vasoconstrictor peptides, such as Ang II or endothelin-1, the physiological action of NEPI in vivo depends on the effects of such vasodilating and/or vasoconstricting peptides.  Actually, the effect of Cx, i.e., NEPI, on hemodynamics is controversial. Several reports have documented no change, a decrease or an increase in blood pressure in normotensive and/or hypertensive human subjects in response to NEPI [19-23].  In the present study, Cx produced no hemodynamic changes while it suppressed the increase in aldosterone concentration that was seen in control dogs.  This indicates that Cx did not affect the hemodynamics, at least in this short-term electrical remodeling model, and the suppressive effect of Cx on AERP shortening is not considered to result from its effect on hemodynamics.

### Suppressive effect of Cx on electrical remodeling

In this study, Cx suppressed AERP shortening caused by rapid pacing in comparison with the control.  Therefore, NEPI is considered to prevent atrial electrical remodeling, at least in its early phase.  As the level of ANP was significantly higher in the Cx group than the control, this result seems compatible with our previous work, which documented the suppressive effect of hANP on the early phase of atrial electrical remodeling [6].

The mechanism of the suppressive effect of Cx (NPEI) on electrical remodeling is uncertain, but the signaling of cyclic GMP may play an important role.  Several reports have documented that natriuretic peptides, including ANP and C-type, would increase intracellular cyclic GMP resulting in the accelerated phosphorylation of phospholamban [24-27]. This activates calcium-ATPase in the sarcoplasmic reticulum (SERCA), and the re-absorption of calcium to the sarcoplasmic reticulum (SR) increases, resulting in reduced intracellular calcium overload. As calcium overload is considered an important key to initiate electrical remodeling, especially in its early phase, this mechanism may explain the suppressive effect of Cx on AERP shortening in our model. Although the increased degree of the plasma and atrial tissue cyclic GMP after the rapid pacing were smaller than that of ANP, they were increased in Cx group as compared to control group.  This might be the reason that the level of cyclic GMP, i.e., second messenger depends on not only ANP but also other factors.

### Effect of Cx on the renin-angiotensin-aldosterone system

There are some reports which have indicated that suppression of the renin-angiotensin-aldosterone system (RAAS) may prevent atrial fibrillation, mainly through the reduction of myocardial fibrosis [18,28]. As Cx (NEPI) also inhibits the degradation of active-renin or Ang II, it may show an inverse effect on preventing the construction of the atrial fibrillation substrate.  Although there was no significant difference in this study, the plasma level of Ang II was increased in comparison with that of active-renin in the Cx group before the pacing.  The mechanism of this change was unknown, but did not affect the hemodynamics at least. On the other hand, the plasma level of aldosterone was even higher in the control group than the Cx group after rapid pacing.  Some of these changes might be affected by the inhibitory action of ANP on the renin-angiotensin-aldosterone system [29,30], the release of aldosterone from adrenal [30], or the expression of CYP11B2 (aldosterone synthetase) [31]. Meanwhile, the effect of Cx on the renin-angiotensin system in the myocardium was not obvious in our study, which might become another key in long-term electrical and structural remodeling.

## Limitations

There are several limitations in our study.  First, the number of experiments is relatively small.  However in this study, the dogs were carefully chosen, basing on the single species (not mongrel), same gender, similar body weight and age, so that the difference between individual should be small, therefore we think we can conclude the results of this study basing on this number.  Second, because pharmacological autonomic blockade was not used in this study, its influence on electrical remodeling could not be excluded.  It is unclear whether the autonomic nerve system is related to atrial electrical remodeling, but intravenous ANP in patients with a decompensated heart has been demonstrated to benefit cardiac sympathetic nerve activity and improve left ventricular remodeling [32].  Third, the inhibitory effect of Cx on long-term electrical remodeling is unknown.  Because ANP has additional functions, i.e., improvement of hemodynamics by natriuresis and suppression of RAAS besides reduction of calcium overload such as verapamil, these effects might suppress the structural remodeling in chronic AF.  Since it is unknown how does Cx influence RAAS and the atrial electrical remodeling in long term AF model, this points will be evaluated with and without angiotensin-converting enzyme inhibitor in addition to Cx in the next study.  Fourth, we did not evaluate the electrophysiological properties in the left atrium. This evaluation was omitted mainly because of study time limitation during relatively quick recovery from the electrical remodeling with short-term, i.e., 7 hour pacing. Finally, this study is principally the observation of the effect of Cx on the electrical remodeling in specific experimental condition.  Although several parameters have been changed simultaneously, the increases in ANP and cyclic GMP were compatible for the effect of Cx and the prevention of calcium overload.  According to these results, we speculated the increases in ANP and cyclic GMP as the explanation of the observation.  To demonstrate our hypothesis more clearly, further study in which increased cyclic GMP acts independently for the suppression of electrical remodeling should be examined.

## Conclusion

Cx, a NEPI, suppressed AERP shortening in a canine rapid atrial pacing model with no significant change in hemodynamics.  As the levels of plasma ANP, plasma and atrial tissue cyclic GMP were higher in the Cx group than the control, the suppressive effect of Cx on electrical remodeling might be appeared through the reduction of calcium overload through of enhanced SERCA activity.  This finding may lead to a new therapeutic modality to prevent the construction of an arrhythmogenic substrate, especially in patients with heart failure.

## Figures and Tables

**Table 1:**
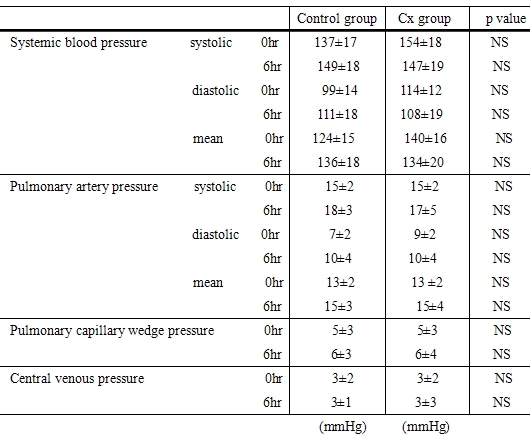
Changes of hemodynamic findings
